# *c*-3,*t*-3-Dimethyl-*r*-2,*c*-6-diphenyl­piperidin-4-one

**DOI:** 10.1107/S1600536809041580

**Published:** 2009-10-17

**Authors:** M. Thenmozhi, S. Ponnuswamy, J. Umamaheshwari, M. Jamesh, M. N. Ponnuswamy

**Affiliations:** a Centre of Advanced Study in Crystallography and Biophysics, University of Madras, Guindy Campus, Chennai 600 025, India; b Department of Chemistry, Government Arts College (Autonomous), Coimbatore 641 018, India

## Abstract

In the title compound, C_19_H_21_NO, the piperidine ring adopts a chair conformation. The two phenyl rings attached to the piperidine ring at 2 and 6 positions occupy equatorial orientations and the dihedral angle between them is 57.53 (11)°. In the crystal, the mol­ecules are connected *via* weak inter­molecular C—H⋯π inter­actions, leading to a zigzag chains.

## Related literature

For general background to piperidine derivatives, see: Badorrey *et al.* (1999 ▶); Nalanishi *et al.* (1974 ▶); Elena *et al.* (2002 ▶). For hybridization, see: Beddoes *et al.* (1986 ▶). For hydrogen-bond motifs, see: Bernstein *et al.* (1995 ▶). For ring conformational analysis, see: Cremer & Pople (1975 ▶); Nardelli (1983 ▶). For the synthesis of the title compound, see Noller & Baliah (1948 ▶).
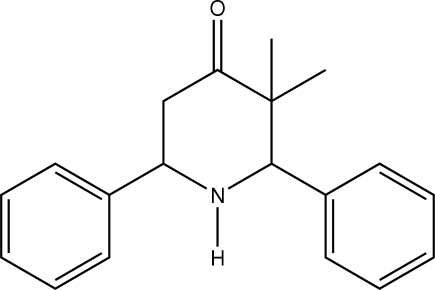


## Experimental

### 

#### Crystal data

C_19_H_21_NO
*M* *_r_* = 279.37
Triclinic, 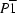

*a* = 6.0293 (4) Å
*b* = 10.8198 (6) Å
*c* = 12.1649 (6) Å
α = 98.559 (2)°
β = 92.836 (3)°
γ = 96.677 (3)°
*V* = 777.62 (8) Å^3^
*Z* = 2
Mo *K*α radiation
μ = 0.07 mm^−1^
*T* = 293 K
0.20 × 0.20 × 0.18 mm

#### Data collection

Bruker Kappa APEXII area-detector diffractometer
Absorption correction: multi-scan (*SADABS*; Sheldrick, 2001 ▶) *T*_min_ = 0.986, *T*_max_ = 0.987
15310 measured reflections
3556 independent reflections
1930 reflections with *I* > 2σ(*I*)
*R*_int_ = 0.036

#### Refinement

*R*[*F* ^2^ > 2σ(*F* ^2^)] = 0.050
*wR*(*F* ^2^) = 0.168
*S* = 1.06
3556 reflections
197 parameters
1 restraint
H atoms treated by a mixture of independent and constrained refinement
Δρ_max_ = 0.18 e Å^−3^
Δρ_min_ = −0.19 e Å^−3^

### 

Data collection: *APEX2* (Bruker, 2004 ▶); cell refinement: *SAINT* (Bruker, 2004 ▶); data reduction: *SAINT*; program(s) used to solve structure: *SHELXS97* (Sheldrick, 2008 ▶); program(s) used to refine structure: *SHELXL97* (Sheldrick, 2008 ▶); molecular graphics: *ORTEP-3* (Farrugia, 1997 ▶); software used to prepare material for publication: *SHELXL97* and *PLATON* (Spek, 2009 ▶).

## Supplementary Material

Crystal structure: contains datablocks global, I. DOI: 10.1107/S1600536809041580/bt5058sup1.cif

Structure factors: contains datablocks I. DOI: 10.1107/S1600536809041580/bt5058Isup2.hkl

Additional supplementary materials:  crystallographic information; 3D view; checkCIF report

## Figures and Tables

**Table 1:** Hydrogen-bond geometry (Å, °)

*D*—H⋯*A*	*D*—H	H⋯*A*	*D*⋯*A*	*D*—H⋯*A*
C10—H10⋯*Cg*3^i^	0.93	2.95	3.648	133

Symmetry codes: (i) 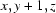. *Cg*3 is the centroid of the C15–C20 ring.

**Fig. 1.**
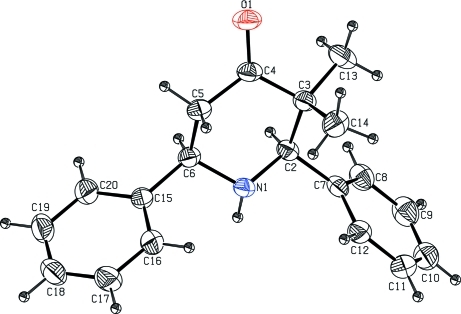
The ORTEP plot of the molecule with 30% probability displacement ellipsoids.

**Fig. 2.**
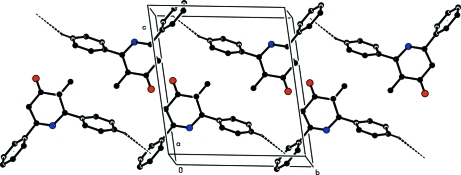
The crystal packing of the molecules viewed along b - axis.

## supplementary crystallographic information

### Comment

Various piperidine derivatives are present in numerous alkaloids (Badorrey *et al.*, 1999). Piperidines have been found to exhibit blood cholesterol-lowering activities (Nalanishi *et al.*, 1974). Trans-platinum piperidine derivatives deserve evaluation of their efficacy in tumor-bearing animals (Elena *et al.*, 2002). In view of these importance, the crystal structure of the title compound has been carrried out.

The ORTEP plot of the molecule is shown in Fig. 1. The piperidine ring adopts chair conformation and the ring-puckering parameters (Cremer & Pople, 1975) are: q_2_ = 0.1578 (20)Å, q_3_ = -0.5364 (21)Å, and φ = 176.7 (8)°, and the smallest asymmetry parameter Δ_s_(N1)=Δ_s_(C4) = 1.97 (16)° (Nardelli, 1983). The two phenyl rings attached to the piperidine ring at 2,6- positions occupy equatorial orientation [C7-C2-C3-C4 = -174.77 (16)°; C4-C5-C6-C15 = 175.09 (16)°], respectively and the dihedral angle between them is 57.52 (11)°. The methyl groups attached at position 3 of the piperidine ring takes up syn-periplanar [C13-C3-C4-O1 = -22.3 (3)°] and anti-clinical [C14-C3-C4-O1 = 97.1 (2)°] orientations. The sum of the bond angles at N1[329.62 (5)°] of the piperidine ring is in accordance with *sp*^3^ hybridization (Beddoes *et al.*, 1986).

The molecules are connected via intermolecular C–H···π interactions (Table 1) which lead to a zig–zag chain running along *b * – axis in addition to van der Waals forces (Fig. 2).

### Experimental

The procedure reported by Noller and Baliah was followed for the preparation of this compound (Noller & Baliah, 1948). Benzaldehyde (21ml), 3-methyl-2-butanone (10ml) and ammonium acetate (8gm) were dissolved in distilled ethanol (50ml) and heated over boiling water bath with shaking, until an yellow colour developed and changed into orange. The solution was left undisturbed for 14 hours. The solid thrown out was filtered, purified and recrystallized from ethanol.

### Refinement

The H atom bonded to N was freely refined. H atoms bonded to C were positioned geometrically (C-H = 0.93 - 0.98 Å) and allowed to ride on their parent atoms, with *U*_iso_(H) = 1.5*U*_eq_(C) for methyl H and 1.2*U*_eq_(C) for other H atoms. The components of the anisotropic displacement parameters of C18 and C19 in the direction of the bond between them were restrained to be equal within an effective standard deviation of 0.001.

### Crystal data

**Table:**

C_19_H_21_NO	*Z* = 2
*M**_r_* = 279.37	*F*(000) = 300
Triclinic, *P*1	*D*_x_ = 1.193 Mg m^−^^3^
Hall symbol: -P 1	Mo *K*α radiation, λ = 0.71073 Å
*a* = 6.0293 (4) Å	Cell parameters from 3556 reflections
*b* = 10.8198 (6) Å	θ = 1.7–28.2°
*c* = 12.1649 (6) Å	µ = 0.07 mm^−^^1^
α = 98.559 (2)°	*T* = 293 K
β = 92.836 (3)°	Block, colourless
γ = 96.677 (3)°	0.20 × 0.20 × 0.18 mm
*V* = 777.62 (8) Å^3^	

### Data collection

**Table:**

Bruker Kappa APEXII area-detector diffractometer	3556 independent reflections
Radiation source: fine-focus sealed tube	1930 reflections with *I* > 2σ(*I*)
graphite	*R*_int_ = 0.036
ω and φ scans	θ_max_ = 28.2°, θ_min_ = 1.7°
Absorption correction: multi-scan (*SADABS*; Sheldrick, 2001)	*h* = −8→7
*T*_min_ = 0.986, *T*_max_ = 0.987	*k* = −14→13
15310 measured reflections	*l* = −15→15

### Refinement

**Table:**

Refinement on *F*^2^	Secondary atom site location: difference Fourier map
Least-squares matrix: full	Hydrogen site location: inferred from neighbouring sites
*R*[*F*^2^ > 2σ(*F*^2^)] = 0.050	H atoms treated by a mixture of independent and constrained refinement
*wR*(*F*^2^) = 0.168	*w* = 1/[σ^2^(*F*_o_^2^) + (0.0729*P*)^2^ + 0.1205*P*] where *P* = (*F*_o_^2^ + 2*F*_c_^2^)/3
*S* = 1.06	(Δ/σ)_max_ = 0.014
3556 reflections	Δρ_max_ = 0.18 e Å^−^^3^
197 parameters	Δρ_min_ = −0.19 e Å^−^^3^
1 restraint	Extinction correction: *SHELXL97* (Sheldrick, 2008), Fc^*^=kFc[1+0.001xFc^2^λ^3^/sin(2θ)]^-1/4^
Primary atom site location: structure-invariant direct methods	Extinction coefficient: 0.020 (5)

### Special details

**Table:**

Geometry. All esds (except the esd in the dihedral angle between two l.s. planes) are estimated using the full covariance matrix. The cell esds are taken into account individually in the estimation of esds in distances, angles and torsion angles; correlations between esds in cell parameters are only used when they are defined by crystal symmetry. An approximate (isotropic) treatment of cell esds is used for estimating esds involving l.s. planes.
Refinement. Refinement of F^2^ against ALL reflections. The weighted R-factor wR and goodness of fit S are based on F^2^, conventional R-factors R are based on F, with F set to zero for negative F^2^. The threshold expression of F^2^ > 2sigma(F^2^) is used only for calculating R-factors(gt) etc. and is not relevant to the choice of reflections for refinement. R-factors based on F^2^ are statistically about twice as large as those based on F, and R- factors based on ALL data will be even larger.

### Fractional atomic coordinates and isotropic or equivalent isotropic displacement parameters (Å^2^)

**Table:**

	*x*	*y*	*z*	*U*_iso_*/*U*_eq_	
C2	0.1988 (3)	0.24655 (18)	0.32290 (15)	0.0425 (5)	
H2	0.0400	0.2403	0.3379	0.051*	
C3	0.3320 (3)	0.22876 (19)	0.43103 (15)	0.0468 (5)	
C4	0.2694 (3)	0.0952 (2)	0.45372 (16)	0.0493 (5)	
C5	0.2448 (4)	−0.00903 (19)	0.35615 (16)	0.0524 (6)	
H5A	0.1674	−0.0845	0.3778	0.063*	
H5B	0.3923	−0.0271	0.3353	0.063*	
C6	0.1161 (3)	0.02397 (18)	0.25591 (15)	0.0442 (5)	
H6	−0.0376	0.0336	0.2750	0.053*	
C7	0.2596 (3)	0.37123 (18)	0.28453 (15)	0.0455 (5)	
C8	0.1269 (4)	0.4668 (2)	0.30562 (18)	0.0588 (6)	
H8	0.0030	0.4548	0.3472	0.071*	
C9	0.1733 (5)	0.5791 (2)	0.2668 (2)	0.0723 (7)	
H9	0.0818	0.6421	0.2825	0.087*	
C10	0.3532 (5)	0.5982 (2)	0.2053 (2)	0.0750 (8)	
H10	0.3836	0.6738	0.1781	0.090*	
C11	0.4897 (5)	0.5055 (2)	0.18330 (19)	0.0704 (7)	
H11	0.6138	0.5187	0.1421	0.084*	
C12	0.4420 (4)	0.3928 (2)	0.22252 (17)	0.0551 (6)	
H12	0.5344	0.3301	0.2069	0.066*	
C13	0.2732 (5)	0.3230 (2)	0.52803 (18)	0.0740 (8)	
H13A	0.1152	0.3097	0.5367	0.111*	
H13B	0.3139	0.4072	0.5133	0.111*	
H13C	0.3535	0.3117	0.5952	0.111*	
C14	0.5846 (4)	0.2435 (2)	0.41959 (19)	0.0644 (7)	
H14A	0.6588	0.2207	0.4839	0.097*	
H14B	0.6361	0.3294	0.4137	0.097*	
H14C	0.6176	0.1895	0.3540	0.097*	
C15	0.1096 (4)	−0.07639 (18)	0.15558 (16)	0.0460 (5)	
C16	0.2873 (4)	−0.0837 (2)	0.08964 (18)	0.0605 (6)	
H16	0.4134	−0.0243	0.1061	0.073*	
C17	0.2818 (5)	−0.1775 (2)	−0.0003 (2)	0.0728 (7)	
H17	0.4041	−0.1810	−0.0438	0.087*	
C18	0.0987 (5)	−0.2653 (2)	−0.0261 (2)	0.0726 (7)	
H18	0.0952	−0.3285	−0.0872	0.087*	
C19	−0.0791 (5)	−0.2598 (2)	0.0383 (2)	0.0732 (7)	
H19	−0.2041	−0.3199	0.0214	0.088*	
C20	−0.0753 (4)	−0.1654 (2)	0.12890 (19)	0.0620 (6)	
H20	−0.1982	−0.1621	0.1720	0.074*	
N1	0.2245 (3)	0.14438 (15)	0.23353 (13)	0.0435 (4)	
O1	0.2492 (3)	0.07213 (16)	0.54724 (12)	0.0687 (5)	
H1	0.168 (4)	0.1575 (19)	0.1717 (19)	0.058 (7)*	

### Atomic displacement parameters (Å^2^)

**Table:**

	*U*^11^	*U*^22^	*U*^33^	*U*^12^	*U*^13^	*U*^23^
C2	0.0418 (11)	0.0477 (12)	0.0367 (10)	0.0071 (9)	0.0038 (8)	0.0012 (8)
C3	0.0453 (12)	0.0570 (13)	0.0363 (10)	0.0018 (9)	0.0019 (8)	0.0053 (9)
C4	0.0431 (12)	0.0697 (15)	0.0359 (11)	0.0030 (10)	0.0002 (8)	0.0143 (10)
C5	0.0626 (14)	0.0528 (13)	0.0438 (11)	0.0061 (10)	0.0021 (10)	0.0155 (10)
C6	0.0457 (12)	0.0475 (12)	0.0408 (10)	0.0046 (9)	0.0043 (8)	0.0118 (9)
C7	0.0534 (13)	0.0460 (12)	0.0343 (10)	0.0066 (9)	−0.0041 (8)	−0.0013 (8)
C8	0.0655 (16)	0.0525 (14)	0.0571 (13)	0.0139 (11)	−0.0024 (11)	0.0016 (11)
C9	0.093 (2)	0.0524 (15)	0.0708 (16)	0.0209 (13)	−0.0092 (15)	0.0045 (12)
C10	0.113 (2)	0.0494 (15)	0.0612 (15)	0.0044 (15)	−0.0152 (15)	0.0155 (12)
C11	0.094 (2)	0.0629 (16)	0.0531 (14)	−0.0037 (14)	0.0060 (12)	0.0151 (12)
C12	0.0680 (15)	0.0488 (13)	0.0489 (12)	0.0079 (11)	0.0092 (10)	0.0065 (10)
C13	0.096 (2)	0.0789 (17)	0.0410 (12)	0.0077 (14)	−0.0001 (12)	−0.0051 (12)
C14	0.0462 (14)	0.0788 (17)	0.0677 (15)	−0.0021 (12)	−0.0080 (10)	0.0215 (13)
C15	0.0554 (13)	0.0428 (11)	0.0402 (10)	0.0039 (9)	−0.0023 (9)	0.0113 (9)
C16	0.0711 (16)	0.0504 (13)	0.0572 (14)	0.0011 (11)	0.0117 (12)	0.0015 (11)
C17	0.096 (2)	0.0644 (16)	0.0565 (14)	0.0135 (14)	0.0155 (13)	−0.0003 (12)
C18	0.110 (2)	0.0549 (14)	0.0486 (14)	0.0124 (15)	−0.0144 (11)	0.0005 (11)
C19	0.0874 (18)	0.0583 (15)	0.0652 (15)	−0.0101 (13)	−0.0227 (10)	0.0058 (12)
C20	0.0625 (15)	0.0627 (15)	0.0569 (14)	−0.0055 (12)	−0.0061 (11)	0.0103 (11)
N1	0.0562 (11)	0.0423 (10)	0.0321 (9)	0.0055 (8)	0.0016 (7)	0.0071 (7)
O1	0.0743 (11)	0.0921 (12)	0.0396 (8)	−0.0042 (9)	0.0012 (7)	0.0223 (8)

### Geometric parameters (Å, °)

**Table:**

C2—N1	1.458 (2)	C10—H10	0.9300
C2—C7	1.504 (3)	C11—C12	1.380 (3)
C2—C3	1.555 (3)	C11—H11	0.9300
C2—H2	0.9800	C12—H12	0.9300
C3—C4	1.520 (3)	C13—H13A	0.9600
C3—C13	1.525 (3)	C13—H13B	0.9600
C3—C14	1.528 (3)	C13—H13C	0.9600
C4—O1	1.209 (2)	C14—H14A	0.9600
C4—C5	1.499 (3)	C14—H14B	0.9600
C5—C6	1.522 (3)	C14—H14C	0.9600
C5—H5A	0.9700	C15—C16	1.372 (3)
C5—H5B	0.9700	C15—C20	1.377 (3)
C6—N1	1.457 (2)	C16—C17	1.372 (3)
C6—C15	1.504 (3)	C16—H16	0.9300
C6—H6	0.9800	C17—C18	1.362 (4)
C7—C12	1.381 (3)	C17—H17	0.9300
C7—C8	1.382 (3)	C18—C19	1.360 (4)
C8—C9	1.371 (3)	C18—H18	0.9300
C8—H8	0.9300	C19—C20	1.383 (3)
C9—C10	1.361 (4)	C19—H19	0.9300
C9—H9	0.9300	C20—H20	0.9300
C10—C11	1.374 (4)	N1—H1	0.85 (2)
			
N1—C2—C7	109.70 (15)	C10—C11—C12	119.8 (2)
N1—C2—C3	109.78 (16)	C10—C11—H11	120.1
C7—C2—C3	114.76 (15)	C12—C11—H11	120.1
N1—C2—H2	107.4	C11—C12—C7	121.2 (2)
C7—C2—H2	107.4	C11—C12—H12	119.4
C3—C2—H2	107.4	C7—C12—H12	119.4
C4—C3—C13	109.95 (17)	C3—C13—H13A	109.5
C4—C3—C14	106.01 (17)	C3—C13—H13B	109.5
C13—C3—C14	110.43 (18)	H13A—C13—H13B	109.5
C4—C3—C2	108.99 (15)	C3—C13—H13C	109.5
C13—C3—C2	109.16 (18)	H13A—C13—H13C	109.5
C14—C3—C2	112.25 (16)	H13B—C13—H13C	109.5
O1—C4—C5	120.6 (2)	C3—C14—H14A	109.5
O1—C4—C3	121.75 (19)	C3—C14—H14B	109.5
C5—C4—C3	117.57 (16)	H14A—C14—H14B	109.5
C4—C5—C6	112.35 (17)	C3—C14—H14C	109.5
C4—C5—H5A	109.1	H14A—C14—H14C	109.5
C6—C5—H5A	109.1	H14B—C14—H14C	109.5
C4—C5—H5B	109.1	C16—C15—C20	118.2 (2)
C6—C5—H5B	109.1	C16—C15—C6	121.47 (18)
H5A—C5—H5B	107.9	C20—C15—C6	120.3 (2)
N1—C6—C15	111.07 (15)	C17—C16—C15	121.0 (2)
N1—C6—C5	107.32 (16)	C17—C16—H16	119.5
C15—C6—C5	111.89 (17)	C15—C16—H16	119.5
N1—C6—H6	108.8	C18—C17—C16	120.4 (2)
C15—C6—H6	108.8	C18—C17—H17	119.8
C5—C6—H6	108.8	C16—C17—H17	119.8
C12—C7—C8	117.4 (2)	C17—C18—C19	119.4 (2)
C12—C7—C2	121.77 (19)	C17—C18—H18	120.3
C8—C7—C2	120.70 (19)	C19—C18—H18	120.3
C9—C8—C7	121.6 (2)	C18—C19—C20	120.5 (2)
C9—C8—H8	119.2	C18—C19—H19	119.8
C7—C8—H8	119.2	C20—C19—H19	119.8
C10—C9—C8	120.0 (3)	C15—C20—C19	120.4 (2)
C10—C9—H9	120.0	C15—C20—H20	119.8
C8—C9—H9	120.0	C19—C20—H20	119.8
C9—C10—C11	119.9 (2)	C6—N1—C2	111.51 (15)
C9—C10—H10	120.0	C6—N1—H1	107.9 (14)
C11—C10—H10	120.0	C2—N1—H1	111.3 (15)
			
N1—C2—C3—C4	−50.7 (2)	C7—C8—C9—C10	0.4 (4)
C7—C2—C3—C4	−174.77 (16)	C8—C9—C10—C11	−0.8 (4)
N1—C2—C3—C13	−170.78 (17)	C9—C10—C11—C12	0.8 (4)
C7—C2—C3—C13	65.1 (2)	C10—C11—C12—C7	−0.5 (3)
N1—C2—C3—C14	66.4 (2)	C8—C7—C12—C11	0.0 (3)
C7—C2—C3—C14	−57.6 (2)	C2—C7—C12—C11	176.84 (19)
C13—C3—C4—O1	−22.3 (3)	N1—C6—C15—C16	39.6 (3)
C14—C3—C4—O1	97.1 (2)	C5—C6—C15—C16	−80.3 (2)
C2—C3—C4—O1	−141.9 (2)	N1—C6—C15—C20	−141.7 (2)
C13—C3—C4—C5	160.91 (19)	C5—C6—C15—C20	98.4 (2)
C14—C3—C4—C5	−79.7 (2)	C20—C15—C16—C17	−0.3 (3)
C2—C3—C4—C5	41.3 (2)	C6—C15—C16—C17	178.5 (2)
O1—C4—C5—C6	139.4 (2)	C15—C16—C17—C18	0.2 (4)
C3—C4—C5—C6	−43.7 (2)	C16—C17—C18—C19	−0.3 (4)
C4—C5—C6—N1	53.0 (2)	C17—C18—C19—C20	0.5 (4)
C4—C5—C6—C15	175.09 (16)	C16—C15—C20—C19	0.5 (3)
N1—C2—C7—C12	−41.6 (2)	C6—C15—C20—C19	−178.3 (2)
C3—C2—C7—C12	82.6 (2)	C18—C19—C20—C15	−0.6 (4)
N1—C2—C7—C8	135.14 (19)	C15—C6—N1—C2	170.45 (16)
C3—C2—C7—C8	−100.7 (2)	C5—C6—N1—C2	−67.0 (2)
C12—C7—C8—C9	0.0 (3)	C7—C2—N1—C6	−165.75 (16)
C2—C7—C8—C9	−176.82 (19)	C3—C2—N1—C6	67.3 (2)

### Hydrogen-bond geometry (Å, °)

**Table:**

*D*—H···*A*	*D*—H	H···*A*	*D*···*A*	*D*—H···*A*
C10—H10···Cg3^i^	0.93	2.95	3.648	133

Symmetry codes: (i) *x*, *y*+1, *z*.
